# Foundress numbers and the timing of selective events during interactions between figs and fig wasps

**DOI:** 10.1038/s41598-018-37498-3

**Published:** 2019-03-04

**Authors:** Bao-Fa Sun, Rui-Wu Wang

**Affiliations:** 1 Center for Ecological and Environmental Sciences, Institute of Medical Research, Northwestern Polytechnical University, Xi’an, 710072 China; 2 CAS Key Laboratory of Genomics and Precision Medicine, Beijing Institute of Genomics, Chinese Academy of Sciences, Beijing, 100101 China

## Abstract

In intimate mutualisms between hosts and symbionts, selection can act repeatedly over the development times of the interacting individuals. Although much is now known about the overall ecological conditions that favor the evolution of mutualism, a current challenge is to understand how natural selection acts on the number and kinds of partners to shape the evolution and stability of these interactions. Using the obligate fig-fig wasp mutualism, our experiments showed that the proportion of figs developed to maturity increased quickly to 1.0 as the number of foundresses increased, regardless of whether the foundresses carried pollen. Selection against pollen-free wasps did not occur at this early stage in fig development. Within figs that developed, the proportion of galls producing adult wasps remained high as the number of pollen-carrying foundresses increases. In contrast, the proportion of galls producing adult wasps decreased as the number of pollen-free foundresses increased. Viable seed production increased as the number or proportion of pollen-carrying foundresses increased, but the average number of wasp offspring per pollen-carrying foundress was highest when she was the sole foundress. These results show that figs and their pollinator wasps differ in how fitness effects are distributed throughout the development of the interaction and depend on the number and proportion of pollen-carrying foundresses contributing to the interaction. These results suggest that temporal fluctuations in the local number and proportion of pollen-carrying wasps available to enter figs are likely to have strong but different effects on the figs and the wasps.

## Introduction

Mutualisms between species often involve interactions with multiple partners that may also interact with each other. At one extreme, selection could favor hosts that interact intimately with many individuals of a single clone of a vertically inherited symbiont; at the other extreme, selection could favor individuals that interact briefly with many individuals of a wide range of species, forming a multi-specific coevolving network^1,2^. The timing of selection on mutualistic partners will therefore vary with the ecological form of the mutualism. Although much is now known about the overall ecological conditions that favor the evolution of mutualism, a current challenge is to understand how natural selection acts on the number and kinds of partners to shape the evolution and stability of these interactions due to intrinsic benefit conflict between host and symbiont^3–5^.

Stability in mutualistic interactions is likely to be maintained in a variety of ways at different spatial and temporal scales^1,6^ as selection acts upon each component of an interaction. Hosts may evolve traits that regulate costs relative to benefits, including extreme sanctioning of partners that impose costs that exceed their benefits of the interaction^7–10^. Examples include fruit or root nodules abortion when visited only by the cheating individuals or species, as has been shown in interactions between legume and rhizobia, figs and figs wasp and yuccas and yuccas moths^5,11–14^. Mutualisms, however, may remain stable even through more subtle means, as hosts evolve, for example, to restrict the activities of partners in ways that are less detrimental to host fitness. Examples include restriction in the length of time that a host makes a reward available or restriction of access to the reward itself, as occurs in production of floral rewards by plants^15–18^. In some cases, however, stability may be the result of a combination of selection pressures on hosts and partners that happen to result in mutualism as selection favors participants on each side of the interaction that maximize their own fitness^5,19^.

The interactions between plants and pollinating floral parasites provide opportunities to evaluate how fitness in each participant is built up throughout the course of an interaction from the time of encounter to the production of seeds and the next generation of adult pollinators. These interactions occupy a complex middle ground between intimate symbiotic mutualisms and mutualisms among free-living species. More than a thousand plant species worldwide are involved in these mutualisms, which have originated repeatedly among plant families^8,20–23^. The interaction begins as one or more pollen-carrying adult insects lay eggs into the flowers while simultaneously pollinating the flowers^20^. Thereafter the fitness of the developing host plant and that of the developing larvae of pollinators are fully linked. The interaction proceeds through several stages, each of which provides an opportunity for selection to act on the partners.

The interactions between figs and fig wasps are among the most well-known and diverse mutualisms found in nature, and they have persisted for millions of years^24,25^. In these interactions, one or more pollen-carrying fig wasps enter a receptive fig and one or more pollen-free fig wasps may also enter the fig.^4,26^, but the proportion of pollen free fig wasps is likely to be low (e.g., 0–5% in several neotropical fig species)^11^. The pollinating wasps usually firstly disperse the pollen for the fig and then deposit their eggs into the nucellus of fig flowers. The nucellus of fig flowers swell rapidly, which is probably initiated by the ovipositing female^27^. A subsequent development of the endosperm, which may be due to double fertilization^12^ or maybe parthenogenetic induced by the larva^28^, will feed the wasp larva. The number of seeds produced by the fig and the number of adult wasps emerging from the fig fruit depend on the subsequent sequence of life history events within the fig.^5,11,26,29^. Each of those events provides an opportunity for selection to favor figs that have been entered by few or many fig wasps.

We evaluated how selection may act on wasps and figs throughout the development of a fig fruit to control the number and ratio of pollen-carrying and pollen-free adult fig wasps that enter figs. We experimentally tested the hypothesis that selection should favor (1) pollen-carrying fig wasps that lay eggs in figs entered by other pollen-carrying wasps, due to that the host tree abort less fruits when more seeds are produced^5,17^, (2) pollen-free fig wasps that lay eggs in figs with few other pollen-free fig wasps, avoiding more intensive host sanction^18^, and (3) figs that selectively reduce the number of adult wasps emerging from figs containing many pollen-free fig wasps. Specifically, we evaluated the fate of figs and fig wasps for figs receiving between 1 and 9 pollen-carrying fig wasps, between 1 and 9 pollen-free fig wasps, or a ratio between0:9 to 9:0 pollen-carrying to pollen-free figs wasps.

## Materials and Methods

### Study site

The study was carried out in and near the Xishuangbanna Tropical Botanic Garden (XTBG) in Yunnan province, China (21°41′N, 101°25′E). This garden is approximately 600 m above sea level and has a monsoonal climate. The rainy and dry seasons last from May to October and from November to April, respectively. Mean annual precipitation is 1,557 mm with about 80% occurring during the rainy season^30^. The mean annual temperature is 21 °C, and the mean annual relative humidity is 87%. All experiments were performed between May and September of 2011.

### Study species

*Ficus racemosa* is widely distributed from India to Australia^31^ and resides in the Sycomorus section of *Ficus*. *Ficus racemosa* is a monoecious fig and it is actively pollinated by *Ceratosolen fusciceps*^31^. *Ficus racemosa* is usually a large free-standing tree (up to 25 m in height). It produces large crops of large spherical figs (up to approximately 45 mm in diameter when mature), which grow on short branches (racemes) that are attached to the trunk and larger branches. The flowering patterns of *F. racemosa* are typical for monoecious Ficus, with intra-tree synchrony and inter-tree asynchrony^21^. Both wasps and seeds are produced in each fig resulting in a clear conflict between the mutualists as during seed and larval development, especially when the unutilized common resource is limited as a result of foundresses increase^32^. Seed production, however, is only one component of plant fitness. In order to reproduce through spread of both female and male gametes, a tree must produce seeds and also wasps for pollen dispersal. In contrast, wasps only need to produce more wasps and they do so by galling some host flowers.

## Methods

We used *Ficus racemosa* and its pollinator wasps *Ceratosolen fusciceps* to assess the effects on fitness of the number of wasps carrying pollen relative to number not carrying pollen. The experiments used two categories of wasps. Pollinating wasps carried pollen to the figs in pollen pockets that they had collected naturally as they emerged from their natal figs; pollen-free wasps were prevented from carrying pollen by collecting specimens from treated syconia in which we removed the stamen before the adult wasp emerged from the galled flowers. This manipulation of syconia to prevent wasps from collecting pollen has been successfully used in previously studies^5,10^.

In one set of experiments, we introduced either pollen-free (P−) or pollen-carrying (P+) wasps into receptive fruits. In these experiments, we conducted five treatments in which either 1, 3, 5, 7 or 9 foundresses were introduced. The foundresses were sequentially introduced into each receptive syconium within two days, allowing a 2 hour time interval between introductions of each wasp when the total foundress number was high. Some data from these experiments has been used in our previous paper that addressed a different set of questions^10,18^. In another set of experiments, we introduced 9 wasps, but the number of pollen-carrying wasps within that total varied from 0, 2, 4, 6, 8 to 9. The sample size of each treatment is larger than 20. Collectively, these experiments allowed an evaluation of a wide range of ecologically and evolutionarily relevant patterns of colonization of figs by fig wasp foundresses and the responses of figs under all these scenarios.

In all experiments, we selected 2–4 trees from two sample sites of locally fragmented and highly fragmented forest. When the treated syconia developed to maturity, but before the pollinator wasps cut exit holes, we collected the syconia and injected a solution of 75% ethanol, killing the present adult wasps. We opened each synconium, cut open each gall, and counted the viable seeds, and male wasps, and female wasps. We also cut open each gall to ensure that the results also included any well-developed adult wasps that had not yet emerged from their natal gall. Mature syconia had empty galls that contained no adult wasps. Such galls may result when females successfully oviposited but larvae failed to develop^10,12^.

Statistics analysis: SPSS 17.0 was used to conduct all the statistics. The fig size is an independent parameter and therefore is included as covariate. The sample site (fragmented or forested) and tree are also included as a covariate and GLM or one way ANOVA are used in regression analysis and mean comparison analysis. We use Pearson correlation in the correlation analysis.

## Results

### Fig and wasp production mediated through the number of foundresses

**Figure 1:**
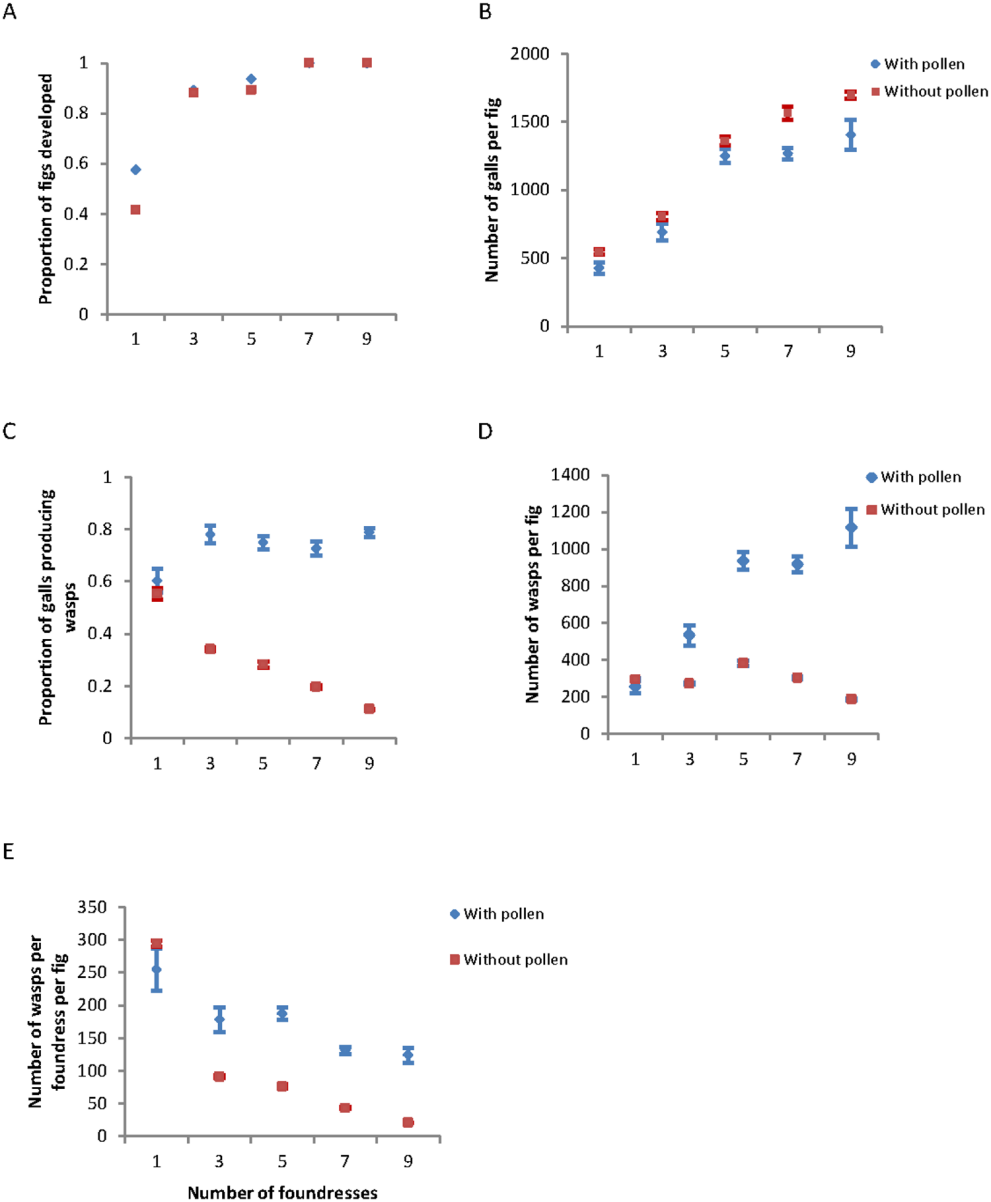
Fig and wasp production relative to the number of pollen-carrying (blue) or pollen-free (red) fig wasps that entered a fig. In these experiments, all foundresses in a fig were either pollen-carrying or pollen-free fig wasps. (**A**) The proportion of fig synconia (inflorescences) that developed into mature figs; (**B**) the number of galls produced within each fig; (**C**) the proportion of galls that produced adult wasps; (**D**) the number of adult wasps that emerged from a mature fig: (**E**) the number of adult wasps produced per foundress from a fig. Values are means ± 1 standard deviation. Statistical results for all figures are reported in the Table S1.

The proportion of figs that developed to maturity increased quickly to 1.0 as the number of foundresses increased in both experiments with P+ wasps (Logarithmic regression: R^2^ = 0.94, *F* = 46.201, *df*_*1,3*_, *P* < 0.01) and P− wasps (Logarithmic regression: R^2^ = 0.91, *F* = 32.064, *df*_*1,3*_, *P* < 0.05), and the fig abortion ratio is similar between every P+ and P− treatments (Fig. 1A). In fact, fig development was stimulated by the oviposition activities of the wasps, regardless of whether the fig flowers were pollinated. Hence, selection against pollen-free wasps did not occur at this early stage in fig development.

The number of galls in each fig also increased significantly as the number of foundresses increased in both experiments with P + wasps (quadratic: R^2^ = *0.95, F* = 75.057, *df*_2,98_, *P* < 0.001)and P− wasps (quadratic: R^2^ = *0.886, F* = 380.770, *df*_*2,98*_, *P* < 0.001), regardless of whether the wasps carried pollen (Fig. 1B). This result suggests that there was little interference between wasps during this stage of the interaction. Within figs that developed, the proportion of galls producing adult wasps remained high as the number of pollinating foundresses increased significantly (N = 101, r = 0.30, *P* < 0.01) (Fig. 1C). In contrast, the proportion of galls producing adult wasps decreased significantly as the number of pollen-free foundresses increased (N = 101, r = 0.92, *P* < 0.001). This, then, was the first developmental stage at which selection acted against P− foundresses.

The total number of adult wasps produced by a fig increased significantly as the number of pollinating foundresses increased up to seven wasps (N = 101, r = 0.73, *P* < 0.001) (Fig. 1D). This pattern may result from the greater number of seeds in figs founded by multiple P + wasps (Oneway ANOVA *F*_*4, 96*_ = 23.073, P < 0.001). In contrast, the total number of adult wasps remained low in figs containing only P− foundresses, regardless of the number of foundresses (*F*_*4,96*_ = 63.31, *P* < 0.001). A different pattern was evident, however, when the values were scaled to the number of adult wasps produced per foundress within each fig. A pollen-carrying or pollen-free fig wasp produced significantly more offspring when she was the only foundress (P−: *F*_*4,96*_ = 8.22, *P* < 0.001; P + : *F*_*4,96*_ = 1415.83, *P* < 0.001) (Fig. 1E). In figs entered by more than one wasp, the number of wasps produced per foundress was similar for 3–9 wasps for P + wasps (n = 80, r = 0.164, *P* < 0.001) but decreased linearly for P− wasps (n = 81, r = 0.95, *P* < 0.001). However, when only visited by one foundress, the fig develop similar proportion galls containing the adult wasps regardless whether the foundress carry the pollen or not (*t* = 1.06, *df* = 38, *P* = 0.30). Hence, selection favors wasps that are single foundresses and acts most strongly against pollen-free wasps that enter figs with many other pollen-free wasps.

### Fig and wasp production mediated through galls

**Figure 2:**
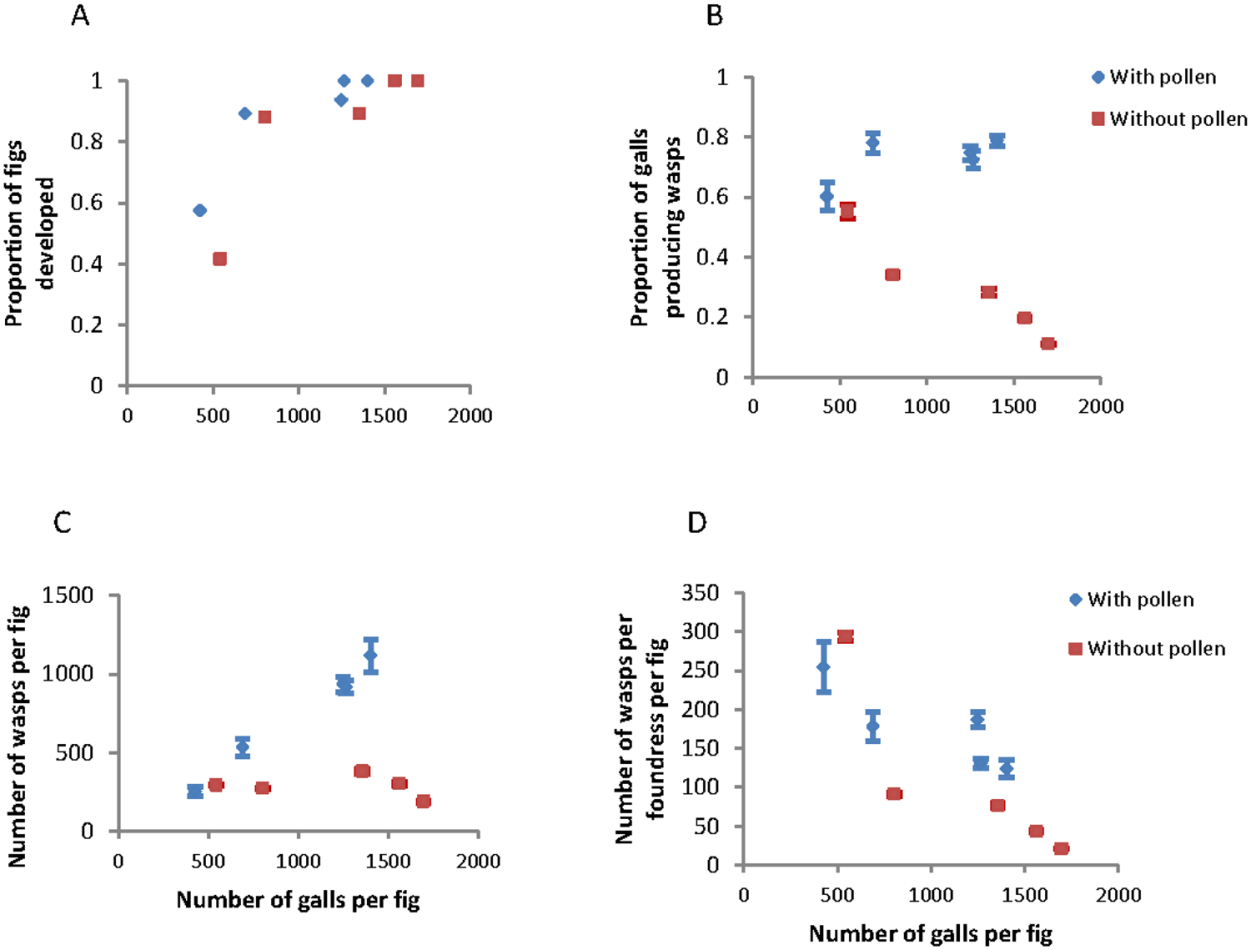
Fig and wasp production relative to the number of galls produced by a fig. Values are for pollen-carrying (blue) or pollen-free (red) fig wasps. Values combine the experiments in which either 1 to 9 pollinating or non-pollinating wasps entered a fig. (**A**) Proportion of fig synconia that developed into mature figs; (**B**) proportion of galls that produced adult wasps; (**C**) number of adult wasps produced from each mature fig; (**D**) number of adult wasps produced per foundress from each mature fig. Values are means ± 2 standard error.

We explored further the relationships between the number of foundresses and the number of adult wasp offspring by evaluating how the effects are mediated through production of galls. The proportion of fig syconia producing mature figs increased rapidly and significantly to 1.0 as the number of galls per fig increased, regardless of whether foundresses carried pollen (Fig. 2A). That is, fig trees did not automatically abort fig syconia with many galls. The proportion of galls producing adult wasps remained high as the number of P + wasps entering a fig increased, but the proportion decreased rapidly and significantly for P− wasps (N = 101, r = 0.916, *P* < 0.001) (Fig. 2C,B). Hence, selection would act most strongly against P− fig wasps that entered a fig with many other P− fig wasps and induced many galls. The same pattern was evident for the number of adult wasps produced per fig (Fig. 2C). That result is reinforced if the values are scaled to the number of adult offspring wasps produced on average per foundress wasp, but pollinating wasps also suffered lower offspring production in figs with many galls (Fig. 2D).

Overall, the sequence of fig and wasp developmental stages, mediated by the number of foundresses and the number of galls, resulted in a marked difference in the number of adult wasps produced by figs containing pollinating wasps as compared with figs containing pollen-free wasps. Pollen-carrying and pollen-free foundresses produced most offspring per foundress when they entered syconia alone. Pollen-free foundress wasps, however, suffered consistently lower production of offspring at all levels of foundress numbers or gall numbers.

### Figs with P+ and P− foundresses

**Figure 3:**
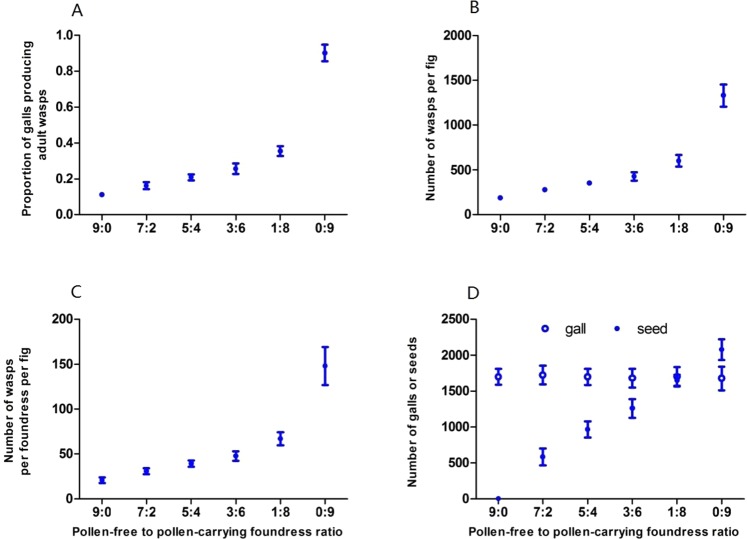
Fig and wasp development relative to the proportion of foundress that are pollen-carrying wasps. All experiments had nine foundresses. (**A**) Proportion of fig synconia that developed into mature figs; (**B**) number of gall produced per fig; (**C**) proportion of galls that produced adult wasps; (**D**) number of wasps per fig. (**E**) Number of wasps per foundress per fig. (**F**) Total number of galls (white bars) and seeds (hatched bars) produced by figs with nine foundresses. Values are means ± 2 standard error.

In the experiments introducing 9 foundresses into each fig, all fig syconia produced mature figs, regardless of the ratio of P− to P+ foundresses (Fig. 3A). The number of galls per fig was similar across all treatments, indicating there was no significant effect of pollination on gall number (*F*_*5, 145*_ = 0.28, *P* = 0.95) (Fig. 3D). The proportion of galls producing adult wasps increased significantly as the proportion of pollinating foundresses increased (N = 151, r = 0.92, *P* < 0.001) (Fig. 3C). The number of adult wasps produced per fig and also per foundress per fig also increased significantly as the proportion of pollinating foundress increased (N = 151, r = 0.91, *P* < 0.001) (Fig. 3B,E).

The effects of the number of foundress wasps on seed production differed from the effects on wasp production. Figs with nine foundress wasps produced the same number of galls, regardless of the combination of P + and P− foundresses entering the fig (*F*_*5, 145*_ = 0.28, *P* = 0.95), (Fig. 3F). Figs with nine pollen-free wasp foundresses produced no seeds, demonstrating that all pollen had been removed in the P− syconia treatment (Fig. 3F). As the ratio of P− to P+ wasps increased from 1:8 to 9:0, however, the number of seeds produced increased linearly (y = 81.744 + 204.129 × , y = number of seeds, x = number of pollen-carried wasps, Linear:R^2^ = *0.959, F* = 3491.27, *df*_1,149_, *P* < 0.001). Hence, from the perspective of plant fitness, more pollinating foundresses are better than few foundresses, at least up to nine foundresses.

## Discussion

The results suggest that increasing the number of foundress wasps entering figs affects wasp and fig fitness in different ways, at least at the level of individual figs. The interaction between figs, P+ fig wasps, and P− fig wasps favors pollinating fig wasps that enter syconia alone. The number of offspring produced by only one pollinating foundress female is greatest under these conditions (Fig. 1E). In this situation, there is low overall production of galls which may benefit development of the offspring (Fig. 2D). This result might explain why the foundresses with stronger competitive ability are selected for in the process of evolution, because the strong interference among the foundresses^4,33^ and even fight and kill among the foundresses^34^ will decrease the overall egg deposition of foundresses.

The interference, though, is asymptotic. Some direct or indirect interference may occur among small numbers of pollinating foundresses, as shown in the lower number of offspring produced per female per fig in multi-foundress figs. The effects, however, do not continue to amplify as the number of foundresses increases in these experiments (Fig. 1E). In contrast, P− foundresses suffer decreased fitness with each additional P− foundress. This result may occur either through selection that has favored direct sanctioning of figs that have many P− foundresses, or indirectly through physiological mechanisms that withdraw resources from figs with many galls but few developing seeds^12,35^. Pollen-free foundresses may fare best when entering figs with a small number of pollen-carrying foundresses. The mutualism is therefore regulated in part through the effects on the number of wasps produced relative to the number and ratio of P+ and P− foundresses.

In contrast, under the treatments in which fig wasps entered sequentially, figs continue to benefit from having more pollinating foundresses that could disperse more pollen for fig flowers therefore more seed production. The fig could behaviorally encourage more foundresses enter its cavities. If one foundress is in its cavity, the fig could keep its ostiole open to allow additional pollinating wasps entering^33^. However, if wasps enter the fig almost simultaneously, more foundresses might also lead to the interference competition, thereby decreasing both total pollination and oviposition of foundresses^4,33^. In fact, when many foundresses enter the fig’s cavity, the fig will quickly enclose its bract ostiole to prevent more foundresseses from entering fig cavity^33^. Such mechanism could prevent the potential conflict between the figs seed production and pollinator offspring production, because both seed production and wasp offspring must be at the expense of common resource—female flowers of figs^4,36–38^. Selection on figs could therefore act to optimize the number of foundresses that can enter either simultaneously or sequentially^33,39^.

In nature, the number of foundresses entering a fig varies greatly. Fewer than 10 wasps per fig is common, but occasionally figs can have up to 70 wasps^40^. The results reported here therefore represent a realistic number of foundresses. The difference between wasps and figs in the effects of foundress number suggests that the mutualism between wasps and fig wasps may be maintained in part by divergent selection dynamic between fig and fig wasp. Selection on the wasps should favor few foundresses per fig, but selection on the plants should favor more foundresses. The foundress number usually is between 7–9 foundresses in the study population, based on the results of our experiments and observations. The interference competition among foundresses, if foundress number is higher than 9, might lead to a decrease of production of galls and seeds^4,33^.

When P+ and P− wasps enter fig cavities together, the fitness effects differ on host figs and the pollinating wasps. Selection on figs should select for more pollinating foundresses. Selection on figs should also favor plants that can distinguish galls produced by pollen-carrying foundresses from those not carrying pollen^11^. In the absence of such targeted sanction by figs.^11^, selection may favor wasps that do not carry pollen if pollen collection and dispersal is costly to fitness.

The combination of antagonistic and mutualistic selection between the host and symbiont might lead to population oscillations among the host, pollinating wasps and cheating wasps or parasitic wasps^5,10,41^. Moreover, evolutionary changes in the ability to distinguish mutualists from cheaters may explain shifts in the evolution and prevalence of parasites and mutualists over the evolutionary time scale in these kinds of interaction^5,42,43^. It may also partly explain the coexistence of parasitic wasps (including the cheating individuals of pollinating wasps and non-pollinating wasps) and cooperative individuals in some plant populations but not necessarily in all populations^13,43–46^.

The results also suggest that figs, P+ wasps, and P− wasps may all differ in the stages of the interaction at which selection acts mostly strongly. As a result selection may favor figs that are able to respond to the number and ratio of P+ and P− wasps entering a fig. Fig trees abort the fruits in which non-pollinating wasps *Sycophaga testacea* oviposit before pollinating wasps^5,29^, and fig trees often also abort fruits only entered by only one foundress. This abortion may be a pre-adaptation mechanism that could save the resource for the fig trees^47,48^. However, fig trees often do not abort fruits in which pollen-free wasps of *Sycophaga mayri* oviposit later than pollinating wasps. Even the female offspring of pollen-free pollinating wasps can contribute to fig fitness because, once they emerge, they could disperse pollen to another tree.

Fig wasps may also diversify their behaviors to maximize their fitness. Selection on wasps could act to separate emergence times and oviposition peaks among individuals within and among species to avoid the competition^5,49–51^. The results reported here also suggest that pollinator wasps might diversify their strategies at the early stages. Even after entering the fig cavities, selection might act on foundresses to avoid ovipositing at the same time, if interference or fruit abortion occurs above a threshold of simultaneous oviposition attempts.

## Conclusion

The experiments here showed that figs and their pollinator wasps differ in how fitness effects are distributed throughout the development of the interaction and depend on the number and proportion of pollen-carrying foundresses contributing to the interaction. The results suggest that selection on the partners may differ in intensity during different stages of a mutualistic interaction. Hence, stability of mutualisms may sometimes be maintained under some ecological conditions, even without direct selection on hosts to sanction partners, through the combined selection pressures acting on each species across the developmental stages of the participants.

## Supplementary information

Supplementary information

## Data Availability

If the paper get published, the data could be available through the online of the journal.
