# Supplementary material for: Foundress numbers and the timing of selective events during interactions between figs and fig wasps
Source: Sci Rep. 2019 Mar 4;9:3420. doi: 10.1038/s41598-018-37498-3 (PMC6399315; doi:10.1038/s41598-018-37498-3)
Supplement: Supplementary file 1 — Supplementary information [file 41598_2018_37498_MOESM1_ESM.doc]

1. Relations between two factors

| Fig | X-factor | Y-factor | Condition | R^2^ | P value | methods |
| --- | --- | --- | --- | --- | --- | --- |
| Fig 1A | Foundress number | Proportion of figs developed | with pollen | 0.731 | 0.065 | Pearson (N=5)  y=0.048x+0.642,F=8.137,df=1,3 |
| Fig 1A | Foundress number | Proportion of figs developed | with pollen | 0.950 | 0.050 | Quadratic  y=-0.011x^2^+0.158x+0.454, F=18.594, df=2,2 |
| Fig 1A | Foundress number | Proportion of figs developed | without pollen | 0.703 | 0.076 | Pearson (N=5)  y=0.064x+0.516,F=0.703,df=1,3 |
| Fig 1A | Foundress number | Proportion of figs developed | without pollen | 0.915 | 0.085 | Quadratic  y=-0.015x^2^+0.214x+0.261, F=10.757, df=2,2 |
| Fig 1B | Foundress number | Number of galls | with pollen | 0.657 | 0.000 | Pearson (N=100)  y=131.790x+456.890,F=187.488,df=1,98 |
| Fig 1B | Foundress number | Number of galls | with pollen | 0.697 | 0.000 | Quadratic  y=-13.802x^2^+269.808x+222.260, F=111.599,df=2,97 |
| Fig 1B | Foundress number | Number of galls | without pollen | 0.862 | 0.000 | Pearson (N=100)  y=153.175x+428.925,F=611.515,df=1,98 |
| Fig 1B | Foundress number | Number of galls | without pollen | 0.886 | 0.000 | Quadratic  y=-10.927x^2^+262.443x+243.170, F=378.581,df=2,97 |
| Fig 1C | Foundress number | Proportion of galls producing adult wasps | with pollen | 0.699 | 0.000 | Pearson (N=100)  y=0.047x+0.517,F=227.775,df=1,98 |
| Fig 1C | Foundress number | Proportion of galls producing adult wasps | with pollen | 0.787 | 0.000 | Quadratic  y=-0.007x^2^+0.119x+0.396, F=179.533,df=2,97 |
| Fig 1C | Foundress number | Proportion of galls producing adult wasps | without pollen | 0.469 | 0.000 | Pearson (N=100)  y=-0.017x+0.302,F=86.653,df=1,98 |
| Fig 1C | Foundress number | Proportion of galls producing adult wasps | without pollen | 0.741 | 0.000 | Quadratic  y=-0.005x^2^+0.038x+0.209, F=138.918,df=2,97 |
| Fig 1D | Foundress number | Number of wasps per fig | with pollen | 0.746 | 0.000 | Pearson (N=100)  y=144.850x+159.685,F=288.314,df=1,98 |
| Fig 1D | Foundress number | Number of wasps per fig | with pollen | 0.768 | 0.000 | Quadratic  y=-10.392x^2^+248.771x-16.981, F=160.401,df=2,97 |
| Fig 1D | Foundress number | Number of wasps per fig | without pollen | 0.109 | 0.001 | Pearson (N=100)  y=9.700x+188.605,F=11.936,df=1,98 |
| Fig 1D | Foundress number | Number of wasps per fig | without pollen | 0.822 | 0.000 | Quadratic  y=-10.507x^2^+114.765x+9.995, F=223.776,df=2,97 |
| Fig 1E | Foundress number | Number of wasps per foundress per fig | with pollen | 0.277 | 0.000 | Pearson (N=100)  y=-11.650x+253.677,F=37.475,df=1,98 |
| Fig 1E | Foundress number | Number of wasps per foundress per fig | with pollen | 0.298 | 0.000 | Quadratic  y=1.370x^2^-25.351x+276.969, F=20.593,df=2,97 |
| Fig 1E | Foundress number | Number of wasps per foundress per fig | without pollen | 0.922 | 0.000 | Pearson (N=100)  y=-11.961x+126.363,F=1160.335,df=1,98 |
| Fig 1E | Foundress number | Number of wasps per foundress per fig | without pollen | 0.932 | 0.000 | Quadratic  y=0.526x^2^-17.221x+135.305, F=665.860,df=2,97 |
| Fig 2A | Gall number | Proportion of figs developed | with pollen | 0.760 | 0.054 | Pearson (N=5)  y= 3.5E-4x+0.490,F=9.498,df=1,3 |
| Fig 2A | Gall number | Proportion of figs developed | with pollen | 0.891 | 0.109 | Quadratic  y=-6.6E-7x^2^+0.002x-0.136, F=8.175,df=2,2 |
| Fig 2A | Gall number | Proportion of figs developed | without pollen | 0.713 | 0.072 | Pearson (N=5)  y= 4.1E-4x+0.345,F=7.443,df=1,3 |
| Fig 2A | Gall number | Proportion of figs developed | without pollen | 0.844 | 0.156 | Quadratic  y=-6.9E-7x^2^-0.002x-0.378, F=5.410,df=2,2 |
| Fig 2B | Gall number | Proportion of galls producing adult wasps | with pollen | 0.481 | 0.000 | Pearson (N=100)  y= 2.38E-4x+0.481,F=90.734,df=1,98 |
| Fig 2B | Gall number | Proportion of galls producing adult wasps | with pollen | 0.540 | 0.000 | Quadratic  y=-1.33E-7x^2^+0.001x-0.317, F=56.919,df=2,97 |
| Fig 2B | Gall number | Proportion of galls producing adult wasps | without pollen | 0.282 | 0.000 | Pearson (N=100)  y= -8.13E-5x+0.307,F=38.452,df=1,98 |
| Fig 2B | Gall number | Proportion of galls producing adult wasps | without pollen | 0.282 | 0.000 | Quadratic  y=-6.16E-9x^2^-6.65E-5x+0.300, F=19.090,df=2,97 |
| Fig 2C | Gall number | Number of wasps per fig | with pollen | 0.945 | 0.000 | Pearson (N=100)  y= 1.002x-234.472,F=1686.111,df=1,98 |
| Fig 2C | Gall number | Number of wasps per fig | with pollen | 0.946 | 0.000 | Quadratic  y=4.87E-5x^2^+0.885x-174.643, F=848.884,df=2,97 |
| Fig 2C | Gall number | Number of wasps per fig | without pollen | 0.194 | 0.000 | Pearson (N=100)  y= 0.080x+148.194,F=23.552,df=1,98 |
| Fig 2C | Gall number | Number of wasps per fig | without pollen | 0.346 | 0.000 | Quadratic  y=-1.12E-4x^2^+0.350x+10.318, F=25.652,df=2,97 |
| Fig 2D | Gall number | Number of wasps per foundress per fig | with pollen | 0.009 | 0.339 | Pearson (N=100)  y= -0.013x+210.123,F=0.925,df=1,98 |
| Fig 2D | Gall number | Number of wasps per foundress per fig | with pollen | 0.121 | 0.002 | Quadratic  y=7.21E-5x^2^-0.187+298.825  F=6.651, df=2,97 |
| Fig 2D | Gall number | Number of wasps per foundress per fig | without pollen | 0.609 | 0.000 | Pearson (N=100)  y= -0.060x+133.274,F=152.885,df=1,98 |
| Fig 2D | Gall number | Number of wasps per foundress per fig | without pollen | 0.690 | 0.000 | Quadratic  y=3.450E-5x^2^-0.143+175.691  F=107.869, df=2,97 |

1. ANOVA test results between groups with and without pollen, and among 5 mixed groups

| Fig | Foundress number | Test factors | df | F | P value |
| --- | --- | --- | --- | --- | --- |
| Fig 1B | 1 | Number of galls | 1,38 | 0.000 | 0.984 |
| Fig 1B | 3 | Number of galls | 1,38 | 0.808 | 0.374 |
| Fig 1B | 5 | Number of galls | 1,38 | 4.301 | 0.045 |
| Fig 1B | 7 | Number of galls | 1,38 | 0.075 | 0.785 |
| Fig 1B | 9 | Number of galls | 1,38 | 6.764 | 0.013 |
| Fig 1C | 1 | Proportion of galls producing adult wasps | 1,38 | 206.058 | 0.000 |
| Fig 1C | 3 | Proportion of galls producing adult wasps | 1,38 | 377.622 | 0.000 |
| Fig 1C | 5 | Proportion of galls producing adult wasps | 1,38 | 919.091 | 0.000 |
| Fig 1C | 7 | Proportion of galls producing adult wasps | 1,38 | 1266.312 | 0.000 |
| Fig 1C | 9 | Proportion of galls producing adult wasps | 1,38 | 5383.436 | 0.000 |
| Fig 1D | 1 | Number of wasps per fig | 1,38 | 56.859 | 0.000 |
| Fig 1D | 3 | Number of wasps per fig | 1,38 | 164.744 | 0.000 |
| Fig 1D | 5 | Number of wasps per fig | 1,38 | 402.219 | 0.000 |
| Fig 1D | 7 | Number of wasps per fig | 1,38 | 219.018 | 0.000 |
| Fig 1D | 9 | Number of wasps per fig | 1,38 | 208.682 | 0.000 |
| Fig 1E | 1 | Number of wasps per foundress per fig | 1,38 | 56.859 | 0.000 |
| Fig 1E | 3 | Number of wasps per foundress per fig | 1,38 | 164.744 | 0.000 |
| Fig 1E | 5 | Number of wasps per foundress per fig | 1,38 | 402.219 | 0.000 |
| Fig 1E | 7 | Number of wasps per foundress per fig | 1,38 | 219.018 | 0.000 |
| Fig 1E | 9 | Number of wasps per foundress per fig | 1,38 | 208.682 | 0.000 |
| Fig3B | Among 5 groups | Number of galls | 5,131 | 0.187 | 0.967 |
| Fig3C | Among 5 groups | Proportion of galls producing adult wasps | 5,131 | 2324.974 | 0.000 |
| Fig3D | Among 5 groups | Number of wasps per fig | 5,131 | 176.971 | 0.000 |
| Fig3E | Among 5 groups | Number of wasps per foundress per fig | 5,131 | 176.971 | 0.000 |
| Fig3F | Among 5 groups | Number of seeds | 5,131 | 1071.891 | 0.000 |
